# Molly Vollman Makris: Public housing and school choice in a gentrified city: youth experiences of uneven opportunity: Palgrave Macmillan, New York, Palgrave Studies in Urban Education, 2015, p 238, ISBN: 9781137429155

**DOI:** 10.1007/s10901-016-9502-9

**Published:** 2016-02-09

**Authors:** W. R. Boterman

**Affiliations:** Faculty of Social and Behavioural Sciences, Urban Geographies, University of Amsterdam, Amsterdam, The Netherlands

This book addresses the important issue of how gentrification may positively affect the lives and opportunities of lower class residents that manage to stay in a gentrified area. There is a wide body of literature that demonstrates that the positive effects of gentrification on lower class long-term residents are limited at best, and detrimental at worst. Many studies show that meaningful interaction between lower class and middle class residents is scarce and relations are tectonic (Butler and Robson 2001): they may live close to each other but rarely truly interact because they have different everyday practices. One of the few locations where different classes, and for that matter different ethnicities, could potentially meet are day cares and schools. Schools may therefore both offer real potential for the transfer of resources from middle class children to lower class kids. On the other hand, exactly because schools are sites of encounter, they are at the forefront of middle class disaffiliation strategies.

The book investigates the case of the city of Hoboken, close to New York City, which has become thoroughly gentrified. Hoboken, a majority upper middle class area, has an interesting demographic composition. As with most gentrifying and gentrified areas there are clear differences in age cohorts between new and old residents. Hoboken has a clear majority of college-educated residents, of whom most have no children or only very young kids. Among the school-aged kids of the area, however, a (slight) majority is non-white and a substantial part lives in poverty. This stark contrast is the backdrop of this study. By studying primarily the experiences of disadvantaged youth this book fits into a small but expanding literature that focuses on the “other side” of gentrification. Where most gentrification and school choice studies investigate the difficulties and dilemma’s op gentrifiers that have to deal with neighbourhood diversity (Boterman 2013), this book usefully addresses how it is for a disadvantaged (black and Hispanic) minority to live in an expensive majority-white city.

It provides a thoroughly empirical analysis of the school choice context of Hoboken through a mix of methods, including ethnographic observations, semi-structured interviews and focus groups, from a viewpoint of a resident-scholar. Being a resident of Hoboken herself the researcher also draws on personal experiences to demonstrate how school choices are differentiated across class and race. The detailed and careful analysis provides a nuanced and empirically rich account of the dilemma’s and practices of—primarily disadvantaged—parents and children.

The city of Hoboken is a choice-district in which parents have the opportunity to choose between three public elementary schools as well as between three charter schools which are, contrary to most of these schools throughout American cities, attended primarily by advantaged, white children. Vollman Makris demonstrates that charter schools are founded, administrated and attended by advantaged white families and as such “cream off” good students, which reduces the potential benefits of social mix at the district public schools. In fact the author describes some of the mechanisms by which school segregation is produced in the context in which social mix could demographically at least be achieved rather easily.

Vollman Makris argues that “public housing residents opt for their local school, as their school choice decisions are constrained and based primarily on convenience and social networks”. To the contrary, advantaged residents “make these decisions [for charter schools, *WRB*] based on social networks, the reputation of the school, parental involvement and the presence of a clear rigorous educational philosophy”. This reconfirms existing knowledge found by previous studies of school choice and school segregation that demonstrate that middle class, white parents choose different schools than lower class parents in the same neighbourhood. Some emphasise that this is rooted in middle class habitus, prioritising the accumulation of cultural capital through the educational system; others stress that it is mainly an issue of resources and constraints. The case study of this book does not provide much insight into resolving this theoretical issue. Moreover, the research design does not allow for a robust comparison of advantaged and less advantaged children and their parents (something the author also acknowledges in the introduction). By deliberately oversampling disadvantaged kids and parents and only incorporating a small number of white advantaged parents the author does not seem to aim for a comprehensive, relational approach to understanding processes behind school segregation.

The merit of this study lies primarily in the fact that it zooms in on the experiences of disadvantaged youth and on the school choice decisions of these groups which are quite under-researched in both gentrification and school choice studies. By offering a detailed account in which disadvantaged youth get a voice of how they experience the gentrified city of Hoboken, this book contributes to our understanding of how it is to live in a predominantly white middle class area as a minority low class non-white. The book demonstrates that the youngsters in Hoboken have quite a strong feeling of belonging, not just in their public housing area, but in most parts of the city. Many of the middle class community’s amenities are also at the disposal of, and are used by disadvantaged youth. Preschools are perhaps the most clear example of high-quality facilities that are to the benefit of many, including also disadvantaged families. Her work thus provides some ammunition for scholars and policy makers that argue that gentrification is a tide that lifts all boats. However, the most important amenity that could facilitate social mobility and social integration: schools, remain highly segregated. The nearly fully gentrified city of Hoboken still has a highly segregated school landscape, due to a combination of spatial concentration of public housing within Hoboken and school choice opportunities to evade local ethnic and social diversity. Here, low class non-white kids do not take advantage of living in an area overflowing with economic and cultural capital. This is an important lesson for policy makers that see the social mixing/gentrification of disadvantaged neighbourhoods as a solution to school segregation and for social mobility more generally.
